# Telemedicine Utilization Patterns and Implications Amidst COVID-19 Outbreaks in Thailand Under Public Universal Coverage Scheme

**DOI:** 10.1177/00469580241246466

**Published:** 2024-04-27

**Authors:** Nyi-Nyi Zayar, Nitichen Kittiratchakool, Thanayut Saeraneesopon, Rukmanee Butchon, Saudamini Vishwanath Dabak, Patiphak Namahoot, Tanasak Kaewchompoo, Pritaporn Kingkaew, Yot Teerawattananon, Wanrudee Isaranuwatchai

**Affiliations:** 1 Health Intervention and Technology Assessment Program, Ministry of Public Health, Nonthaburi, Thailand; 2 Prince of Songkla University, Hat Yai, Thailand; 3 National Health Security Office, Bangkok, Thailand; 4 National University of Singapore, Singapore; 5 University of Toronto, Toronto, Canada

**Keywords:** telemedicine, pandemics, non-communicable diseases, mental health, interrupted time series analysis

## Abstract

During COVID-19 pandemic, telemedicine was a strategy to facilitate healthcare service delivery minimizing the risk of direct exposure among people. In Thailand, the National Health Security Office has included telemedicine services under the Universal Coverage Scheme to support social distancing policies to reduce the spread of COVID-19. This study aimed to determine the patterns of telemedicine service use during major COVID-19 outbreaks including Alpha, Delta, and Omicron in Thailand. We retrospectively analyzed a dataset of telemedicine e-claims from the National Health Security Office, which covers services reimbursed under the Universal Coverage Scheme between December 2020 and August 2022. An interrupted time-series analysis, Pearson correlation analysis and binary logistic regression were performed. Almost 70% of the patients using telemedicine services were over 40 years old. Most patients used services for mental health problems (25.6%) and major noncommunicable diseases, including essential hypertension (12.6%) and diabetes mellitus (9.2%). The daily number of using telemedicine service was strongly correlated with the number of COVID-19 new cases detected. An immediate change in the trend of using telemedicine was detected at the onset of outbreaks along with the surge of infection. The follow-up use of telemedicine services was not substantial among female, older adults patients and those with non-communicable diseases except mental health problems, and infectious diseases. Strategies need to be developed to reinforced healthcare resources for telemedicine during the surge of outbreaks and sustain the use of telemedicine services for chronic and infectious diseases, regardless of the pandemic, and promote the efficiency of healthcare systems.

**What do we already know about this topic?**
Telemedicine is one of the options to facilitate healthcare service delivery while minimizing the risk of contracting COVID-19 infection around the world.
**How does your research contribute to the field?**
Exploring the trend of using telemedicine services during the different outbreaks of COVID-19 would support future planning on how telemedicine can be incorporated into the current healthcare system, especially during imminent outbreaks and pandemics in a resource-limited setting such as a developing country.
**What are your research’s implications toward theory, practice, or policy?**
This study will help answer policy-relevant questions, such as the most common diseases using the telemedicine service, demographic groups with the highest utilization, and patterns in which the telemedicine services are utilized.

## Introduction

Telemedicine is a branch of telehealth that focuses solely on clinical healthcare delivery over a distance.^1^ Telemedicine services are restricted to clinical evaluation, diagnosis, and treatment of patients, while telehealth covers the broader aspect of both clinical, non-clinical and educational services.^1^ The development of telemedicine systems started with the use of interactive television in 1960s.^2^ Telemedicine is regarded as a solution for healthcare systems with limited resources accompanied with increasing burdens of non-communicable diseases and aging population.^3^ Telemedicine improves the accessibility to health service and financial protection, which are 2 major pillars of Universal Health Coverage.^4^ The interest in telemedicine has exploded at the beginning of the novel coronavirus disease (COVID-19) pandemic in 2020.

During the COVID-19 pandemic, people not infected with coronavirus infection, especially those with chronic ailments such as non-communicable diseases (NCDs) like cardiovascular disease (CVD), diabetes mellitus (DM), chronic respiratory diseases, cancer, mental health problems, and pregnancy, required routine follow-up care without the risk of contracting COVID-19 in the hospital.^5^ On the other hand, the global pandemic led to sudden disruption of healthcare services due to restrictions of public transport, insufficient healthcare personnel due to reassignment to COVID-19 units, and shortage of essential medicines and resources.^6^ The outbreaks of Delta and Omicron variants of coronavirus created further disparity in the healthcare system due to higher rates of transmission than their predecessors.^7,8^ The pandemic affected healthcare services in Thailand as well. Routine healthcare services were affected, and people were hesitant to visit health facilities.^9^ Consequently, telemedicine has become a solution to facilitate healthcare service delivery while minimizing the risk of direct exposure among people.

The pandemic has spurred the global utilization of telemedicine services. In United States (US), the use of telemedicine services increased from 13 000 to 1.7 million visits per week after the spread of the pandemic.^10^ A study done Sichuan Province in China reported that the use of telemedicine among physician increased from 34.1% to 96.6% during the pandemic.^11^ A previous study in Japan reported that the pattern of using telemedicine services differed by age. Patients over 65 years old used telemedicine services 3.25 times more than pre-pandemic, whereas patients under 65 years old used it 2.48 times more than pre-pandemic.^12^ However, there is scarcity of information regarding the use of telemedicine service during the pandemic in Thailand. Moreover, there is limited information on the impact of COVID-19 variant outbreaks on the use of telemedicine services within Thailand and globally.

In Thailand, there are 3 main public health insurance schemes under Universal Health Coverage (UHC): Universal Coverage Scheme (UCS), Civil Servant Medical Benefit Scheme, and Social Security Insurance.^13^ The majority (75%) of the population is covered by UCS.^14^ During the pandemic, the National Health Security Office (NHSO) in Thailand included telemedicine services under UCS to support the social distancing policy and reduce the risk of contact and spread of infection.^15^ The first version of telehealth services In Thailand was implemented since satellite technology began to be used.^16^ The program was initiated by the private sector and witnessed collaboration between the public and private sectors. In 2022, the National Broadcasting and Telecommunication Commission (NBTC) and the Ministry of Public Health aim to adopt and integrate telehealth services into existing healthcare infrastructure.^17^ During the pandemic, Thailand had relatively low number of COVID-19 cases in 2020 but experienced surges in cases nearly at the end of 2020.^18^ To strengthen the public health infrastructure and keep provision of healthcare services to the community in respond to the pandemic, the NHSO introduced telemedicine services as part of public health program at the end of 2020. The telemedicine service can be reimbursed under UCS, primarily for patients with stable chronic diseases, such as hypertension, DM, asthma, cancer, and mental illness.^19^

Telemedicine is an option for minimizing the risk of contracting coronavirus infection during the pandemic and also an alternate healthcare delivery system improving the healthcare accessibility and financial protection. In recent years, Governments are developing strategies to expand and fortify the telemedicine services in nationwide scale, recognizing it crucial role in fulfilling the needs patients using these services. However, there is limited information on the predominant types of diseases most frequently consulted through telemedicine services including their follow-up visits. COVID-19 pandemic exemplifies that the impact of the pandemic is not consistent but fluctuated over time, with subsequent outbreaks. The pattern of using telemedicine services would be changing over time following the outbreak patterns. This can be the novelty of the study while previous studies primarily focused on the pattern of using telemedicine during the overall period of the pandemic. In addition, there may be scarcity of this type of research in Low- and Middle-income countries as compared to the studies focusing on High-income countries. Exploring the trend of using telemedicine services during the COVID-19 outbreak would support future planning on how telemedicine can be incorporated into the current healthcare system, especially during imminent outbreaks and pandemics.

Therefore, this study aimed to: (1) identify the background characteristics of telemedicine service users, including patterns and trends of telemedicine services for various types of diseases under UCS; (2) evaluate the impact of different outbreaks (Alpha, Delta, and Omicron variants) on the frequency of use of telemedicine; and (3) factors associated with follow-up use of telemedicine services during the COVID-19 pandemic in Thailand.

## Methods

### Study Design

This retrospective study analyzed telemedicine service utilization of people under the UCS insurance program in Thailand between December 2020 and August 2022.

### Study Population

People in Thailand who used telemedicine services under UCS’s e-Claim program between December 2020 and August 2022 were retrospectively included in the study. The UCS program covers approximately 75% of the population who are not Government employees and Private sector employees in Thailand.^14^ However, the telemedicine service provided during the study period was in the initial phase of the program and covered a certain proportion of people under the UCS program.

### Study Setting

In Thailand, telemedicine services were provided in both public and private healthcare facilities under standalone conditions, implementing a strategic plan by collaboration of both public and private sectors to roll out nationwide telemedicine services.^20^ As the initial phase of the program under the public sector, telemedicine services were provided in hospitals registered in and assessed by the NHSO, including hospitals outside the Ministry of Public Health and central, community, general, and university hospitals in 25 out of 76 provinces in Thailand.

### Data Sources

We used the telemedicine e-Claim dataset from the NHSO, which covers services reimbursed under the UCS program between December 2020 and August 2022. Telemedicine services supported by other insurance programs were excluded. The e-Claim database stores daily records of individual patient information regarding the utilization of telemedicine services. In this study, we used the background data of the patients, including age and sex, daily number of visits, and type of disease identified by the International Classification of Diseases, 10th revision (ICD-10).^21^ Telemedicine services reimbursed under other programs were not included in this dataset. To determine the impact of the Delta and Omicron variant outbreaks, we used publicly available data on the daily number of new COVID-19 cases reported by the WHO.^22^

### Variables

The outcome variable was frequency of telemedicine service use. The use of telemedicine services on weekends and holidays was extremely low and was not considered in this study. Independent variables included sex (male/female), age, and disease type. The patients were divided into the following age groups: <25, 25 to 40, 41 to 60, and >60 years. The types of diseases were stratified based on the chapters of the ICD-10 coding,^21^ including CVD (I00-I99), DM (E10-E14), chronic respiratory diseases (J30-J98), cancers (C00-C97), mental health and behavioral disorders (F00-F99), infectious and parasitic diseases (A00-B99), pregnancy-related healthcare (O00-O99), and other. NCDs were defined according to WHO Health Organization ICD-10 codes.^23^

### Data Analysis

**Figure 1.**
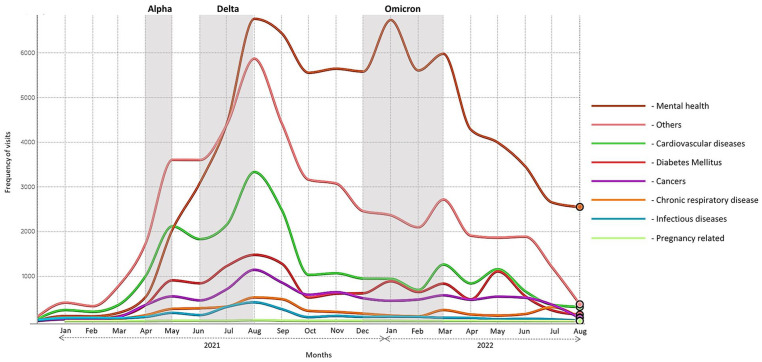
Trends of using telemedicine services during the COVID-19 pandemic stratified by disease type.

Data analysis was performed using R software version 4.1.3.^24^ The background information of the patients using telemedicine services was shown as frequencies and percentages. Chi-square^25^ and Fisher’s exact^26^ tests were used to identify differences between the daily frequency of using telemedicine services by sex and age stratified by disease type. The line graph in the Gapminder tool^27^ was used to display trends in the daily use of telemedicine services by patients over the study period (Figure 1).

Interrupted time series (ITS) analysis^28^ was performed to determine the change in the use of telemedicine services among different age groups (<25, 25-40, 41-60 years, and >60 years) before and during the outbreak of Alpha, Delta, and Omicron variants during the COVID-19 pandemic. We performed the ITS using a segmented negative binomial regression model for each outbreak, as the variance in the frequency of telemedicine visits was larger than the mean value (overdispersion) using the following equation (1).^29^

(1) $$Y_{t}=\beta_{0}+\;\;\beta_{1}T+\;\;\beta_{2}D_{t}+\;\;\beta_{3}T_{t}+e_{t}$$

where $Y_{t}$ is the frequency of taking telemedicine services; T is a continuous variable indicating the time in days passed from the start of the observation period; $D_{t}$ is a dummy variable indicating observations collected before (=0) or after (=1) the beginning of Alpha, Delta, or Omicron outbreaks, respectively; and $T_{t}$ is a continuous variable counting the number of days after the outbreak at time t. The model parameter (β) represents the baseline intercept ( $\beta_{0})$ , pre-outbreak slope ( $\beta_{1}$ ), change in trend at the beginning of the outbreak ( $\beta_{2}$ ), and change in slope during the outbreak ( $\beta_{3}$ ). Term $e_{t}$ represents the random error.

We used Durbin-Watson test to check for autocorrelation of the error terms in regression model.^30^ Durbin-Watson values in the range of 1.5 to 2.5 reflects no serious autocorrelation and Newey-West standard errors^31^ was used to account for autocorrelation. The lag period between the beginning of each outbreak and the change in trend was taken into account.^29^ Seasonality could not be tested because the study covered less than 2 years of data with variability.^32^ Three major outbreaks during the COVID-19 pandemic were defined to conduct ITS analysis: Alpha outbreak (April-May 2021), Delta outbreak (June-August 2021), and Omicron outbreak (December 2021-March 2022). The peak period of COVID-19 variants was identified as the period starting from the time point when the daily number of new cases started to increase until the time point when the highest daily number of new cases was reached.^33^ The immediate increase at the beginning of the outbreak and change in trends of using telehealth services during the outbreak were estimated using the rate ratio (RR) compared to the trend during the pre-outbreak period. Counterfactual lines during the post-outbreak period were predicted to approximate the frequency of telemedicine visits in the absence of outbreaks. R software was used to generate the figures.

Pearson correlation analysis^34^ was used to explore the relationship between the daily number of COVID-19 new cases detected and the daily number of using telemedicine services in different age groups. Binary logistic regression analysis^35^ was applied to determine the background characteristics and group of diseases associated with follow-up telemedicine visits. The odds ratio and 95% confidence interval were used to report the regression and a *P*-value less than .05 was considered significant.

## Results

### Background Characteristics of Patients Using Telemedicine Services

A total of 68 960 patients received telemedicine services for a total of 177 266 visits during the study period. More than half of the patients were female (54.1%). Most patients (42.6%) were over 60 years old. In addition, 11 948 patients (17.3%) used telemedicine services for more than 1 type of disease, which were counted as independent observations. Therefore, 80 908 total number of patients used telemedicine services to treat different diseases (See Supplemental Table 1). Subsequently, more than 40% of those registered visited the follow-up visits more than once (See Supplemental Table 1).

Table 1 shows the background characteristics of the patients using telemedicine services based on different types of diseases. The majority of patients (64.4%) used telemedicine services for NCDs, such as mental health problems (25.6%), CVD (18.6%), DM (9.9%), cancers (6.9%), and chronic respiratory diseases (3.4%). Women used telemedicine services for NCDs more often than men, except for mental health problems. Generally, the use of telemedicine services for NCDs was more common in elderly patients. However, more patients under 25 years old used services for mental health problems. Approximately 60% of the patients used telemedicine services once for all types of diseases, except mental health problems, for which 67.5% of users had more than 1 visit.

**Table 1.** Background Characteristics of Patients Using Telemedicine Services by Disease Type.

	All	Cardio-vascular disease	Diabetes mellitus	Chronic respiratory disease	Cancers	Mental health	Infectious diseases	Pregnancy related health problem	Others
	n (%)	n (%)	n (%)	n (%)	n (%)	n (%)	n (%)	n (%)	n (%)
Total number of patients based on type of disease*	80 908 (100.0)	15 086 (18.6)	8002 (9.9)	2726 (3.4)	5601 (6.9)	20 686 (25.6)	1810 (2.2)	62 (0.1)	26 935 (33.3)
Gender									
Male	36 594 (45.2)	6205 (41.1)	2636 (32.9)	1346 (49.4)	1832 (32.7)	12 646 (61.1)	1003 (55.4)	0 (0.0)	10 926 (40.6)
Female	44 314 (54.8)	8881 (58.9)	5366 (67.1)	1380 (50.6)	3769 (67.3)	8040 (38.9)	807 (44.6)	62 (100.0)	16 009 (59.4)
Age group (years)									
Under 25	14 893 (18.4)	279 (1.8)	135 (1.7)	687 (25.2)	423 (7.6)	7764 (37.5)	170 (9.4)	19 (30.6)	5416 (20.1)
25-40	8597 (10.6)	522 (3.5)	279 (3.5)	219 (8.0)	426 (7.6)	4419 (21.4)	400 (22.1)	28 (45.2)	2304 (8.6)
41-60	21 858 (27.0)	4215 (27.9)	2875 (35.9)	628 (23.0)	1978 (35.3)	5142 (24.9)	806 (44.5)	15 (24.2)	6197 (23)
Over 60	35 562 (44.0)	10 070 (66.8)	4713 (58.9)	1192 (43.7)	2774 (49.5)	3361 (16.2)	434 (24.0)	0 (0.0)	13 018 (48.3)
Frequency of visit									
1	46 420 (57.4)	10 086 (66.9)	4807 (60.1)	1827 (67.0)	3692 (65.9)	6719 (32.5)	1374 (75.9)	35 (56.5)	17 880 (66.4)
2	15 159 (18.7)	3113 (20.6)	1877 (23.5)	528 (19.4)	1031 (18.4)	3758 (18.2)	306 (16.9)	12 (19.4)	4534 (16.8)
3	7282 (9.0)	1225 (8.1)	918 (11.5)	241 (8.8)	437 (7.8)	2443 (11.8)	83 (4.6)	7 (11.3)	1928 (7.2)
4-5	6540 (8.1)	534 (3.5)	365 (4.6)	112 (4.1)	289 (5.2)	3493 (16.9)	39 (2.2)	6 (9.7)	1702 (6.3)
>5	5507 (6.8)	128 (0.8)	35 (0.4)	18 (0.7)	152 (2.7)	4273 (20.7)	8 (0.4)	2 (3.2)	891 (3.3)

* The total number of patients was 68 960, and 11 948 of them used telemedicine services for more than 1 type of disease; these were counted as independent observations.

### Timing of Using Telemedicine Services

The use of telemedicine services during weekends and holidays contributed to only 0.4% of total visits.

### Trend of Using Telemedicine Services Stratified by Different Type of Diseases During COVID-19 Pandemic

Figure 1 shows the trend of telemedicine service use among patients stratified by disease type. The use of telemedicine services significantly increased during the Alpha and Delta variant outbreaks; however, only a few fluctuations were observed during the Omicron outbreak, except for mental health problems. Consistent service use for mental health problems was mainly for schizophrenia.

### Top Five Diagnoses With Most Frequent Telemedicine Visits

The top 5 diagnoses of patients using telemedicine services were essential hypertension (12.6%), type 2 DM (9.2%), schizophrenia (6.1%), pervasive developmental disorders (3.1%), and depressive episodes (3.0%), which accounted for one-third of all patients using telemedicine services.

### Interrupted Time Series Analysis on the Frequency of Telemedicine Visits During Alpha, Delta, and Omicron Variant Outbreaks

**Figure 2.**
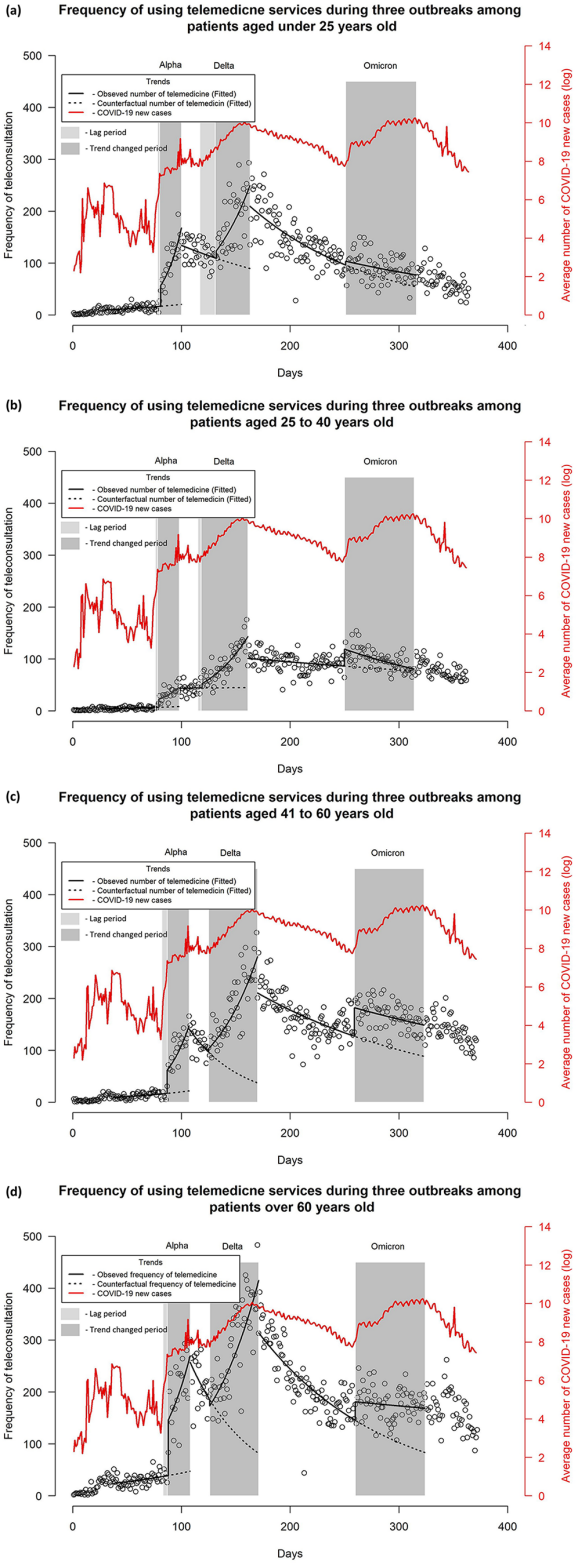
Interrupted time series analysis of the frequency of telemedicine visits during Alpha, Delta, and Omicron variant outbreaks by age: (a) under 25 years old, (b) 25 to 40 years old, (c) 41 to 60 years old, and (d) over 60 years old.

Figure 2 depicts the interrupted time-series analysis of telemedicine service use by age during the Alpha, Delta, and Omicron outbreaks. Overall, a significant immediate increase in used telemedicine service was observed at the onset of Alpha outbreaks across all age groups, and Omicron outbreaks except patients under 25 years old (See Supplemental Table 2). And a significant increase in subsequent trend compared to counterfactual line was detected during Delta outbreaks in all age groups, Alpha outbreaks in those 40 years and below, and Omicron outbreaks in under 25 years and over 60 years old age groups (See Supplemental Table 2). Regarding lag period, a brief lag period of 4 to 5 days was observed after onset of the Alpha outbreaks. At the onset of Delta outbreaks, a longer lag period of 2 weeks was observed in individuals under 25 years of age, while minimal or no lag was observed among the elderly age groups. The trend of daily number of teleconsultations exhibits a comparable trajectory of the daily number of COVD-19 new cases detected from the onset of Alpha until the onset of Omicron outbreaks.

### Correlation Between COVID-19 Daily New Cases and Frequency of Telemedicine Visit

A significant strong correlation was found between the daily number COVID-19 new cases detected and daily number of using telemedicine services during Alpha, Delta until the onset of the Omicron outbreak (Supplemental Table 3).

### Factors Associated With Taking Follow-Up Visits on Telemedicine Services

Table 2 depicts the factors associated with taking follow-up visits on telemedicine services. Being female and older age groups are less likely to take follow-up visits. Compared to other types of diseases, patients with NCDs and infectious diseases were less likely to take follow-up telemedicine visits except those with mental health problems. A higher probability of taking follow-up visits was found during both Delta and Omicron outbreak compared to pre-Alpha outbreak period.

**Table 2.** Factors Associated With Taking Follow-Up Visits on Telemedicine Services.

Characteristics	Adj OR	95% CI	*P*-value
Gender			
Female	Ref	Ref	
Male	1.12	(1.08, 1.16)	<.001
Age group (Years)			
Under 25	Ref	Ref	
25-40	0.80	(0.75, 0.85)	<.001
41-60	0.69	(0.66, 0.73)	<.001
Over 60	0.71	(0.67, 0.75)	<.001
COVID-19 outbreaks			
Pre-Alpha outbreak period	Ref	Ref	
Alpha outbreak period	0.91	(0.80, 1.03)	.128
Delta outbreak period	2.74	(2.46, 3.06)	<.001
Omicron outbreak period	22.48	(20.17, 25.06)	<.001
Group of disease			
Others	Ref	Ref	
Cardiovascular disease	0.65	(0.62, 0.69)	<.001
Diabetes mellitus	0.56	(0.52, 0.60)	<.001
Chronic respiratory disease	0.66	(0.59, 0.75)	<.001
Cancers	0.72	(0.66, 0.78)	<.001
Mental health	1.94	(1.85, 2.02)	<.001
Infectious diseases	0.50	(0.43, 0.59)	<.001
Pregnancy related health problem	1.48	(0.82, 2.68)	.195

Adj OR = adjusted odds ratio; 95% CI = 95% confidence interval; Ref = reference.

## Discussion

This study highlighted the importance of telemedicine during the COVID-19 pandemic. Telemedicine services under UCS program relied on the NHSO’s policy, which primarily focused on major NCDs, such as DM, hypertension, asthma, cancers, mental health, and stable chronic diseases.^19^ In summary, most patients using telemedicine services were over 40 years old. The top 5 most common diseases were essential hypertension, type 2 DM, schizophrenia, pervasive developmental disorders, and depressive episodes. The trend of using telemedicine services was significantly correlated with the daily number of COVID-19 new cases detected. An immediate change in the trend of using telemedicine was detected at the onset of outbreaks along with the surge of infection. More than half of the patients received telemedicine services once. The follow-up use of telemedicine services was poor among female, older age groups and patients with NCDs except mental health problems, and infectious diseases.

Almost half of the patients using telemedicine services during COVID-19 pandemic were over were over 60 years old. This finding is in line with a previous study conducted in Japan, where the mean age of respondents using telemedicine services was over 60 years.^12^ This might be associated with the higher risk of developing chronic diseases, especially NCDs, in the aging population.^36^ Most patients (65%) used telemedicine services for hypertension, DM, and mental health problems. DM and hypertension are the main drivers of NCD prevalence and have shown an increasing trend in Thailand during the past 10 years.^37^ Among the patients who used services for NCDs, women had a higher number of visits, especially for CVD, DM, and cancer, whereas men used more services for mental health problems. Previous study reported that the prevalence of NCDs among women is 2.3 times higher than men, which might be associated with a higher prevalence of low physical activity and overweight/obesity among women.^38^

Among mental health problems, almost half of the services were used for schizophrenia, pervasive developmental disorders, and depressive episodes and used prominently in younger age groups. In previous studies, a positive correlation was found between COVID-19 and risk of developing neurodevelopmental disorders, including schizophrenia and pervasive developmental disorders.^39,40^ The higher use of services for depression aligns with the WHO report indicating a 25% increase in the global prevalence of anxiety and depression during the COVID-19 pandemic related to social isolation, loneliness, fear of contracting infection, loss of family members, and financial constraint.^41^

A notable positive correlation was found between the daily number of telemedicine service use and COVID-19 new cases detected during Alpha and Delta outbreaks. It might be linked to public panic about contracting the infection through in-person healthcare visit, leading to increase use of telemedicine services.^42^ The immediate increase in the use of services was found at the onset of outbreaks, which may related with upsurge of COVID-19 new cases at the onset of outbreak. The biggest change was found during Alpha outbreak, as it was the first outbreak with largest spike in new infections since the beginning of pandemic.^43^ The sudden upsurge in service use would overburden telemedicine service providers, and mitigation measures need to be developed to cope with the demands. On the other hand, additional healthcare resources are required for COVID-19 new cases as well and shifting of resources to COVID-19 department would reduce the quality of care in other healthcare sectors including telemedicine services. Therefore, strategies should be set up to address the increase resources for both telemedicine services and COVID-19 at the onset of outbreak.

However, a substantial increase in the trend after the immediate change was prominent during the Alpha and Delta outbreaks. This might be associated with the fear of contracting these variants due to the increased severity and case fatality rate. The fatality rates of Alpha and Delta variants were 5.3% and 8.56%, respectively.^44,45^ During the Omicron outbreak, the change in the trend was relatively low compared to previous outbreaks, which is consistent with the low severity of the Omicron variant compared to its predecessors. Previous studies have reported that the adjusted hazard ratio of hospital attendance due to Omicron variant infection was 44% lower than that of the Delta variant,^46^ the fatality ratio was approximately 2 times lower than that of delta.^47^ In addition, increased coverage of vaccination during the Omicron outbreak lowered the use of telemedicine services. According to WHO vaccination data, nearly 60% of the Thai population was fully vaccinated at the beginning of the Omicron outbreak.^22^ Therefore, the utilization of telemedicine services may be associated with the severity of the variant and coverage of the vaccination.

In Thailand, nationwide lockdown was implemented during the first outbreak of COVID-19 from April to June 2020. Only partial lockdowns were imposed during Alpha and Delta outbreaks but allow to leave home to visit doctors and no strict preventive measures were imposed during the Omicron outbreak.^48^ Therefore, the impact of lockdown on the utilization of telemedicine services may be less pronounced during the outbreaks.

A consistent trend in the use of mental health services was observed during the Delta and Omicron outbreaks. This was contrary to a finding in Europe that stated that the extent of mental health problems was highest during the first peak months of the pandemic and decreased in the following months.^49^ One of the reasons is that mental health services are concentrated in only the tertiary care level of health facilities (ie, provincial, regional and university hospitals) and around 15 mental health hospitals throughout the Thailand. This finding was in contrary to other health services which are available in every sub-district via health promotion hospitals and district via community hospitals, and posed serious challenges to the Department of Mental Health during the pandemic. As such, the department was actively promoting the use of telemedicine for mental health services and published the guideline for telepsychiatry in 2021.^50^ Another possible reason might be related to the effectiveness of using telemedicine for mental health problems. Previous systematic review and meta-analysis revealed that the medication adherence and treatment outcomes of mental health problems using telemedicine services were comparable to those of in-person services.^51^ Therefore, current telemedicine practices seem to be more favorable for mental health problems than for other types of diseases.

Nearly 60% of the patients sought telemedicine services once, except for mental health problems, where almost 70% of the patients required multiple visits. This reflects the suboptimal utilization of telemedicine services even for chronic diseases, including DM and hypertension, that require regular follow-up visits. Follow-up use of telemedicine services was lower among female, older age groups and those with NCDs except mental health problems and infectious diseases. Previous studies done in US and Iran stated that the use of telemedicine service was poor among female and elderly patients due to poor technology knowledge, distrust in use of technology for healthcare, suboptimal service and concerns about poor clinical outcome.^52,53^ And almost two third of patients with major NCDs including endocrine, CVD, cancers and kidney diseases and those with infectious diseases preferred to use in-person visit over telemedicine.^53^ While telemedicine services provide advantages like cost and time saving, and reduced infection risk, there are room for improvement in terms of system design, administrative support, and ensuring accurate examination and treatment.^53^

In addition, only 0.4% of telemedicine services were sought during weekends and national holidays possibly due to service availability. This finding was contrary to the purpose of providing telemedicine services, which is to improve accessibility to medical care after office hours or for those living in hard-to-reach areas. A study in 2013 conducted in Teladoc, one of the largest telehealth providers in US, reported that 34% of the telemedicine visits were used during weekends and holidays.^54^ Another possible reason for lower utilization of services during weekends and holidays may be related with patient preferences and behavior. Patients may prefer to seek healthcare in-person during non-working days due to perceived urgency or preference for face-to-face interaction with healthcare providers.^53^ While telemedicine aims to enhance accessibility, technical or infrastructure limitations may still exist due to the initial period of implementing telemedicine services. Further mixed-method study will be needed to explore the underlying reasons for poor use of services during holidays, and strategies should be developed to improve the utilization of telemedicine services.

To the best our knowledge, this was the first study to explore the role of telemedicine services in Thailand during the COVID-19 pandemic. Moreover, we explored a series of changes in the use of telemedicine services during major outbreaks of COVID-19 variants. Furthermore, the study analyzed the usage patterns of telemedicine services for different types of diseases stratified by sex and age using a real-world administrative database. This will help answer policy-relevant questions, such as the most common diseases using the service, demographic groups with the highest utilization, and patterns in which the services are utilized. This information is valuable to support the planning of the Universal Digital and Telehealth Coverage Program initiated by the Ministry of Public Health and NBTC. Additionally, in 2024, the Ministry of Public Health (MOPH) in Thailand established a new initiative known as the Smart Energy and Climate Action (SECA) program.^55^ This program promotes the use of telemedicine for patients with NCDs, mental health problems, and respiratory tract infections. The insights from this research findings can be beneficial to the decision-making body of the SECA program to identify these 3 patients’ groups as primary targets and shape the policy direction for telemedicine promotion.

This study had some limitations. The study covered only the population who used telemedicine under UCS’s e-Claim program. Nevertheless, this was the initial phase in the development of a nationwide telemedicine program, and the findings provide information for further planning and development. The study depended on data from when the distribution of services was limited to some hospitals during the initial phase. However, it includes data from a variety of hospitals, such as central, community, general, and university hospitals, which are attributed to major proportions of UCS’s telemedicine programs in Thailand. The duration of the outbreak did not include the declining phase, as the outbreak endpoint could not be identified. However, this covered the period of the major impact of the outbreak, when the highest number of resources was needed. As a nature of analyzing the existing database, the underlying reasons for poor follow-up visits in terms of patients and healthcare sector perspective. A further mixed-method study should be done to explore the complete picture of the pattern of using telemedicine services in Thailand.

Thailand is on track to increase access to healthcare with digital health technologies.^20^ The present findings shed light on how telemedicine can be incorporated into the current healthcare system and how to allocate resources during future pandemics. Further studies are recommended to compare the effectiveness and cost-effectiveness of telemedicine and traditional in-person services and ensure the sustainability of telemedicine services.

## Conclusions

In conclusion, during the COVID-19 pandemic in Thailand, between 2020 and 2022, telemedicine services were predominantly used by patients predominantly with major NCDs who are more likely to be female and individuals aged over 40 years old, as well as those with mental health problems prominent male and those under 25 years old. The demands of telemedicine service correlates to the number of new cases infected during the outbreak of COVID-19 variants with notable severity. In addition, the considerable demands on telemedicine services were also found during the early onset of outbreaks. Healthcare resources for telemedicine services, especially NCDs and mental health problems, should be increased during outbreaks particularly at the early onset. Female, older adults patients and those with NCDs excluding mental health problems, and those with infectious diseases showed fewer use of follow-up telemedicine visits. Promote policy recommendations on provision of community awareness and education programs on technology using telemedicine. Investment in infrastructure, healthcare provider training and technology upgrades to improve examination and treatment accuracy. Integration of telemedicine into routine healthcare delivery for chronic diseases and increase coordination within primary care, specialists and telemedicine platforms. Lastly, strategies for monitoring and evaluation of the existing program and adapting with evolving healthcare need to be developed to sustain the use of telemedicine services for chronic diseases with eligible cases, regardless of the pandemic, and to promote the efficiency of healthcare systems.

## Supplemental Material

sj-docx-1-inq-10.1177_00469580241246466 – Supplemental material for Telemedicine Utilization Patterns and Implications Amidst COVID-19 Outbreaks in Thailand Under Public Universal Coverage Scheme Supplemental material, sj-docx-1-inq-10.1177_00469580241246466 for Telemedicine Utilization Patterns and Implications Amidst COVID-19 Outbreaks in Thailand Under Public Universal Coverage Scheme by Nyi-Nyi Zayar, Nitichen Kittiratchakool, Thanayut Saeraneesopon, Rukmanee Butchon, Saudamini Vishwanath Dabak, Patiphak Namahoot, Tanasak Kaewchompoo, Pritaporn Kingkaew, Yot Teerawattananon and Wanrudee Isaranuwatchai in INQUIRY: The Journal of Health Care Organization, Provision, and Financing
