# Supplementary material for: Telemedicine Utilization Patterns and Implications Amidst COVID-19 Outbreaks in Thailand Under Public Universal Coverage Scheme
Source: Inquiry. 2024 Apr 27;61:00469580241246466. doi: 10.1177/00469580241246466 (PMC11056088; doi:10.1177/00469580241246466)
Supplement: sj-docx-1-inq-10.1177_00469580241246466 – Supplemental material for Telemedicine Utilization Patterns and Implications Amidst COVID-19 Outbreaks in Thailand Under Public Universal Coverage Scheme [file sj-docx-1-inq-10.1177_00469580241246466.docx]

**Supplementary materials**

**Supplementary Table 1.** Background characteristics of patients using telemedicine services based on unique ID, type of diseases and total number of visits.

| **Characteristics** | **Patients with unique ID**  **n (%)** | **Patients used service for different type of diseases**  **n (%)** | **Total number of visits**  **n (%)** |
| --- | --- | --- | --- |
| Number of observations | 68,960 (100.0) | 80,908 (100.0) | 177,266 (100.0) |
| **Gender** |  |  |  |
| Female | 37,337 (54.1) | 44,314 (54.8) | 89,739 (50.6) |
| Male | 31,623 (45.9) | 36,594 (45.2) | 87,529 (49.4) |
| **Age group (years)** |  |  |  |
| Under 25 | 12,707 (18.4) | 14,893 (18.4) | 35,861 (20.2) |
| 25-40 | 7,649 (11.1) | 8,597 (10.6) | 26,616 (15.0) |
| 41-60 | 19,226 (27.9) | 21,858 (27.0) | 49,076 (27.7) |
| Over 60 | 29,378 (42.6) | 35,562 (44.0) | 65,713 (37.1) |
| **Group of disease** |  |  |  |
| Cardiovascular disease | N/A | 15,086 (18 .6) | 23,161 (13.1) |
| Diabetes mellitus | N/A | 8,002 (9.9) | 13,074 (7.4) |
| Chronic respiratory disease | N/A | 2,726 (3.4) | 4,210(2.3) |
| Cancers | N/A | 5,601(6.9) | 9,522 (5.4) |
| Mental health | N/A | 20,686 (25.6) | 75,942 (42.8) |
| Infectious diseases | N/A | 1,810 (2.2) | 2,453 (1.4) |
| Pregnancy related health problem | N/A | 623 (0.1) | 1,201 (0.1) |
| Others | N/A | 26,935 (33.3) | 48,7845 (27.5) |
| Notes: N/A – Not applicable (Patients with unique ID information could not be provided for group of disease because an individual patient can have one or more types of disease and use telemedicine services more than one time for different type of diseases) | | | |

**Supplementary Table 2.** ITS analysis of using telemedicine services during Alpha, Delta and Omicron outbreaks

|  | **Immediate change in trend at the beginning of outbreak,**  **RR (95% CI)** | ***p*-value** | **Change in trend during outbreak,**  **RR (95% CI)** | ***p*-value** |
| --- | --- | --- | --- | --- |
| **Under 25 years old** |  |  |  |  |
| Alpha wave | 3.26 (2.31, 4.60) | **< 0.001** | 1.05 (1.03, 1.07) | **< 0.001** |
| Delta wave | 1.13 (0.92, 1.39) | 0.108 | 1.03 (1.02, 1.04) | **0.025** |
| Omicron wave | 1.07 (0.91, 1.26) | 0.38 | 1.01 (1.01, 1.02) | **0.031** |
|  |  |  |  |  |
| **25 to 40 years old** |  |  |  |  |
| Alpha wave | 3.29 (2.25, 4.81) | **< 0.001** | 1.03 (1.00, 1.05) | **0.039** |
| Delta wave | 1.19 (0.94, 1.50) | 0.145 | 1.02 (1.01, 1.04) | **0.007** |
| Omicron wave | 1.38 (1.23, 1.55) | **< 0.001** | 0.99 (0.98, 0.99) | 0.002 |
|  |  |  |  |  |
| **41 to 60 years old** |  |  |  |  |
| Alpha wave | 3.07 (1.98, 4.74) | **< 0.001** | 1.01 (0.97, 1.04) | 0.767 |
| Delta wave | 1.07 (0.87, 1.30) | 0.537 | 1.04 (1.03, 1.06) | **< 0.001** |
| Omicron wave | 1.43 (1.28, 1.60) | **< 0.001** | 1.00 (1.00, 1.01) | 0.073 |
|  |  |  |  |  |
| **Over 60 years old** |  |  |  |  |
| Alpha wave | 2.99 (1.94, 4.61) | **< 0.001** | 1.01 (0.97, 1.05) | 0.643 |
| Delta wave | 0.93 (0.76, 1.14) | 0.93 | 1.04 (1.02, 1.06) | **< 0.001** |
| Omicron wave | 1.25 (1.11, 1.40) | **0.001** | 1.01 (1.01, 1.01) | **< 0.001** |
| Notes: RR – Rate ratio, CI – Confidence interval | | |  |  |

**Supplementary Table 3.** Correlation coefficient between daily number of COVID-19 new cases and daily frequency of using telemedicine service

| **Frequency of taking telemedicine service** | **Daily number of COVID-19 new cases** | | |
| --- | --- | --- | --- |
|  | Alpha Wave | Delta Wave | Omicron Wave |
| Under 25 years old | **0.79^***^** | **0.73^***^** | -0.10 |
| 25 to 40 years old | **0.69^***^** | **0.89^***^** | -0.12 |
| 41 to 60 years old | **0.73^***^** | **0.85^***^** | 0.15 |
| Over 60 years old | **0.77^***^** | **0.79^***^** | 0.13 |

Note: ^***^ - p value < 0.001
